# Health system challenges in implementing universal health coverage: Asian perspectives and experiences

**DOI:** 10.1186/1472-6963-14-S2-O3

**Published:** 2014-07-07

**Authors:** Tikki Pang, Tiffany Robyn Soetikno, Agus Suwandono

**Affiliations:** 1 Lee Kuan Yew School of Public Policy, National University of Singapore, Singapore; 2 Sarah Lawrence College, Bronxville, New York, USA; 3 School of Public Health, Diponegoro University, Semarang, Indonesia and National Institute of Health Research and Development (NIHRD), Ministry of Health, Jakarta, Indonesia

## 

In line with the global trend towards providing universal health coverage (UHC) as a primary tool in achieving sustainable development in the post-2015, post-MDG era, many low- and middle-income countries in the Asian region are in the midst of developing and implementing various schemes and strategies to achieve UHC. Given the diversity in health system structures, resources and capacities, the implementation of UHC in these countries poses major challenges to health service delivery. Indonesia, the fourth largest country in the world, rolled out its UHC plan, called JKN (National Health Assurance) in early 2014 and faces formidable logistic and administrative challenges with regards to access to medicines, human resources, financing, governance and scaling up health service delivery. Key implementation challenges include those associated with issues of equity, quality and sustainability. The Indonesian experience in rolling out UHC may also be compared to other countries in the region which have implemented UHC with varying degrees of success (e.g. Thailand, Vietnam, Taiwan, India, Malaysia, etc.). In the spirit of ‘reverse innovation’, it is also hoped that lessons learnt from UHC implementation in these countries will provide valuable learning lessons for each other, and for the success of UHC more broadly.

